# Translating Developmental Origins: Improving the Health of Women and Their Children Using a Sustainable Approach to Behaviour Change

**DOI:** 10.3390/healthcare5010017

**Published:** 2017-03-20

**Authors:** Mary Barker, Janis Baird, Tannaze Tinati, Christina Vogel, Sofia Strömmer, Taylor Rose, Rufia Begum, Megan Jarman, Jenny Davies, Sue Thompson, Liz Taylor, Hazel Inskip, Cyrus Cooper, Don Nutbeam, Wendy Lawrence

**Affiliations:** 1 MRC Lifecourse Epidemiology Unit, University of Southampton, Southampton General Hospital, Southampton SO16 6YD, UK; jb@mrc.soton.ac.uk (J.B.); cv@mrc.soton.ac.uk (C.V.); ss@mrc.soton.ac.uk (S.S.); tr@mrc.soton.ac.uk (T.R.); hmi@mrc.soton.ac.uk (H.I.); cc@mrc.soton.ac.uk (C.C.); wtl@mrc.soton.ac.uk (W.L.); 2 NIHR Nutrition Biomedical Research Centre, Southampton Centre for Biomedical Research, Southampton General Hospital, Southampton SO16 6YD, UK; 3 NIHR Dissemination Centre, University of Southampton, Alpha House, Enterprise Road, Southampton SO16 7NS, UK; t.tinati@soton.ac.uk; 4 Formerly of NIHR Nutrition Biomedical Research Centre, Southampton Centre for Biomedical Research, Southampton General Hospital, Southampton SO16 6YD, UK; rb@mrc.soton.ac.uk; 5 Li Ka Shing Centre for Health Research Innovation, Department of Agriculture, Food and Nutritional Science, University of Alberta, Edmonton, AB T7X 5A1, Canada; jarman@ualberta.ca; 6 Formerly of Southampton City Council Public Health Team, Southampton City Council, Civic Centre, Southampton SO14 7LY, UK; Daviesj.1370@gmail.com; 7 Commissioner, Integrated Commissioning Unit, Southampton City Council, Civic Centre, Southampton SO14 7LY, UK; susan.thompson@southampton.gov.uk; 8 Former Senior Commissioning Manager for NHS Southampton, NHS Southampton HQ, Oakley Road, Millbrook, Southampton SO16 4GX, UK; liz.taylor@southampton.gov.uk; 9 Sydney Medical School, Edward Ford Building A27, The University of Sydney, Sydney, NSW 2006, Australia; don.nutbeam@sydney.edu.au

**Keywords:** behaviour change, developmental origins, diet, maternal nutrition, disadvantage

## Abstract

Theories of the developmental origins of health and disease imply that optimising the growth and development of babies is an essential route to improving the health of populations. A key factor in the growth of babies is the nutritional status of their mothers. Since women from more disadvantaged backgrounds have poorer quality diets and the worst pregnancy outcomes, they need to be a particular focus. The behavioural sciences have made a substantial contribution to the development of interventions to support dietary changes in disadvantaged women. Translation of such interventions into routine practice is an ideal that is rarely achieved, however. This paper illustrates how re-orientating health and social care services towards an empowerment approach to behaviour change might underpin a new developmental focus to improving long-term health, using learning from a community-based intervention to improve the diets and lifestyles of disadvantaged women. The Southampton Initiative for Health aimed to improve the diets and lifestyles of women of child-bearing age through training health and social care practitioners in skills to support behaviour change. Analysis illustrates the necessary steps in mounting such an intervention: building trust; matching agendas and changing culture. The Southampton Initiative for Health demonstrates that developing sustainable; workable interventions and effective community partnerships; requires commitment beginning long before intervention delivery but is key to the translation of developmental origins research into improvements in human health.

## 1. Introduction

Translation of the science of Developmental Origins of Health and Disease (DOHaD) into reductions in population risks of non-communicable diseases requires improvements in the nutritional status of girls and young women. Maternal diet and nutritional status are powerful determinants of development in utero and the first years of life [1,2]. To optimise the health and development of babies we therefore need to better support girls and young women to eat more healthily. Women from disadvantaged groups tend to have the poorest quality diets and pregnancy outcomes and should therefore be the focus for this activity [3,4,5,6]. Ecological frameworks explain the confluence of factors that connect disadvantage to poor quality diet. They illustrate the way in which levels of influence affect individual diet, physical activity, and energy balance [7,8]. This places individual behaviour at the heart of interacting layers of surrounding influences, particularly the most immediate family and community characteristics and settings, which are, in turn, embedded in and affected by more distant social, environmental, and policy contexts. A woman’s quality of diet is therefore the product of culture and society, industry, government, her local community, her work, school and peers, her family and her home. Included in this confluence is the woman’s own history and experience of adversity and disadvantage, which have persistent effects on her health [9].

Taking into account this multiplicity of influences suggests that women’s nutritional status could be improved in a number of ways. Supplementing women with individual nutrients may be one way of improving pregnancy outcomes and maternal nutritional status [10,11], Changes in patterns of behaviour and lifestyle are required to improve dietary quality, however, or to support and sustain change in nutritional status. These changes can only be initiated through complex interventions based on an understanding of health behaviour contributed by behavioural science. This paper will describe an example of such an intervention which will take as its focus individual dietary behaviour and how women can be supported to make changes. The paper will then consider challenges to the translation of DOHaD science into health improvement, with reference to work recently carried out in Southampton, UK.

## 2. Changing Health Behaviour

Behavioural science has contributed psychological theory to intervention design and planning, ensuring that interventions have a clear model of how the planned activity might produce the anticipated outcome, and careful description and classification of techniques used in behaviour change interventions, so that successful interventions can be replicated

There is a range of psychological theories that have been used to guide the design and evaluation of complex behaviour change interventions, many of them focusing on the cognitive determinants of individual behaviours [12]. In recent years, however, the focus of interventions to change health behaviour has shifted towards theories that also encompass determinants of behaviour that are less connected to conscious, cognitive processes and more to unconscious, automatic processes or ‘habits’. Socially-constructed behaviours such as eating are the outcome of the two systems, working together synergistically or antagonistically, suggesting that interventions need to address both conscious and unconscious processes to be maximally effective [13]. Recent detailed classification of behaviour change techniques employed in behavioural interventions has produced a taxonomy of approximately a hundred documented techniques, and a method of identifying which of these works best in supporting change in which behaviours and in which populations [14,15]. These techniques can be incorporated into interventions designed using current frameworks that systematically address influences on behaviour at the individual, social and environmental levels using a range of these techniques, accessing both conscious and unconscious cognitive processes [16,17,18,19]. What is missing from both the taxonomy and these frameworks is an effective method of engaging individuals in behaviour change interventions. Descriptions of behaviour change techniques do not include instructions on how best to deliver interventions so as to maximise the likelihood of change in patients and clients.

## 3. Healthy Conversation Skills

‘Healthy Conversation Skills’ is a training programme developed in Southampton UK that provides skills for health and social care practitioners, designed to encourage patient and client engagement in behaviour change. The skills do this by encouraging reflection on habits that patients and clients want to change, thereby making the unconscious conscious and amenable to deliberate change. Key skills are shown in Box 1.

Box 1 **Healthy Conversation Skills.**

**Healthy Conversations: Five Key Skills**Identifying/Creating opportunities—for having a healthy conversation.Asking open discovery questions—‘how’ and ‘what’ questions that lead people to explore their own world and find their own solutions.Listening more than talking—empowering people to identify and take control of their own behaviour change.Reflecting—on practice in order to be more effective.Supporting goal-setting—using SMARTER action planning, staff and women have a sense of change and progress.

These skills were developed in collaboration with local health service commissioners in Southampton UK, who identified that their healthcare staff lacked the confidence to address issues of diet and lifestyle with their clients [20]. At the same time, observation of health and social care practice in the locality identified that opportunities to initiate conversations about behaviour change with clients were frequently missed [21]. Healthy Conversation Skills training recognises that skills to support behaviour change need to go beyond education and instead empower individuals to take control of their health behaviours and to problem-solve. Like Motivational Interviewing and similar approaches, the training offers an approach to supporting behaviour change that understands giving clients information is not enough to change behaviour; clients must also be motivated to change, and have the tools to implement that change [22]. Training in Healthy Conversation Skills is designed to increase self-efficacy and hence build the capacity of practitioners as well as that of patients and clients, and in doing so, change the ethos of those practitioners and their organisations to one that empowers change. In this way, the intervention is intended to operate both at the level of individual behaviour change and on changing the culture of services and, in so doing, trigger automatic as well as reflective processes underlying behaviour change.

The theory underpinning Healthy Conversation Skills is Bandura’s social cognitive theory of the socio-environmental and personal determinants of health [23,24]. The construct of self-efficacy is central to this theory, and describes an individual’s belief that he or she is capable of carrying out a specific behaviour, implying that he or she also has the knowledge and skills to do so. In the Healthy Conversation Skills intervention described in this paper, a woman’s self-efficacy would indicate her belief that she was able to feed herself and her family a healthy diet, because she had both knowledge of healthy eating and confidence and skill to prepare healthy food. Interventions that increase self-efficacy can increase fruit and vegetable consumption [25]. Healthy Conversation Skills are designed to increase self-efficacy through empowering problem-solving, and employ behaviour change techniques intended to support small changes in behaviour, leading to acquisition of mastery skills which Bandura proposes as a means of raising self-efficacy. Taken from the Behaviour Change Technique taxonomy described above, Table 1 describes a sample of the behaviour change techniques that underpin Healthy Conversation Skills and which are used throughout the training. The trainer will, for example, employing the BCT ‘problem-solving’, prompt trainees to identify solutions to their problems supporting change in their patients and clients in the same way that trainees will be encouraging their patients and clients to problem-solve. Trainees demonstrate and practice the skill of using ‘open-discovery’ questions to support this problem-solving activity (Box 1, Skill 2). In the same way, other BCTs are used for specific activities to achieve competence in the five Healthy Conversation Skills. All are incorporated in a way that aligns with the Healthy Conversation Skills’ empowerment philosophy and its aim to enhance self-efficacy in practitioners as well as patients and clients, and are intended to support embedding of skills into practitioners’ routine practice. The full version of Table 1 is available from the authors on request.

Healthy Conversation Skills have been developed to be accessible to all practitioners, whatever their level of experience and role. Trainees have so far included nurses, midwives, play workers, nursery nurses, admin staff, doctors and many types of allied health professionals. The current training model is two four hour sessions a few days apart to groups of around 14–16 people; but it is adaptable and has been delivered in many different formats. Details of the training content have been published elsewhere, and training and evaluation materials are available from the authors on request [27]. The skills are designed to be used opportunistically and are transferable such that they can be used in any situation where practitioners have an opportunity to support behaviour change in their clients. A healthy conversation can be as short as two minutes. The approach is flexible, can be adapted to most situations and can be fitted in to existing ways of working. The intention of the approach is to enhance the effectiveness of existing provision rather than create new structures. Building capacity in the health and social care workforce to support diet and lifestyle change is a sustainable model with the potential to reach disadvantaged populations. If this workforce targets women of child-bearing age and their families, building their behaviour change capacity represents a way of translating findings from the science of DOHaD into health improvement.

## 4. The Southampton Initiative for Health

**Figure 1:**
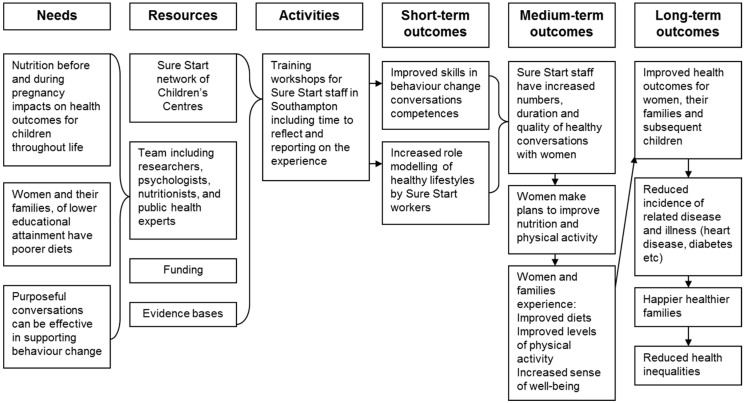
Logic model for the Southampton Initiative for Health, an intervention to train SureStart Children’s Centre staff in Healthy Conversation Skills in order to improve the diets, physical activity levels and well-being of women of child-bearing age.

This model of health improvement was first tested in the Southampton Initiative for Health, an intervention study testing the effectiveness of Healthy Conversation Skills trained health and social care practitioners in improving the self-efficacy, diets and physical activity levels of women with young families in Southampton, UK. The study was designed and run by a partnership between the University of Southampton, the local authority and Public Health in the city. Southampton is a centre for developmental origins, where research with mothers and babies has a relatively high profile. City policy makers and commissioners saw that SureStart Children’s Centres (SSCCs) represented an important opportunity to improve the health and well-being of women of child-bearing age. SSCCs are located in every city across the UK and provide a location for the provision of health and social care services to families with children under five. At the time of launching the Southampton Initiative for Health partnership, SSCCs served the more disadvantaged areas of the city. Southampton is a city in a local authority area which is amongst the top 100 most deprived cities in England, despite being set in an affluent part of the country. Women attending the 14 SureStart Children’s Centres in Southampton therefore represented a population of significant disadvantage [28]. The partnership was developed specifically to target health improvement in this disadvantaged group of women and their families. As the logic model in Figure 1 suggests, health improvements in this group of women were hypothesised to be the outcome of increases in self-efficacy and of eating healthily, engendered by support from SureStart Children’s Centre staff trained in Healthy Conversation Skills. Healthy Conversation Skills training activities were designed to enable staff to learn how to use behaviour change techniques and acquire skills to support these improvements in eating habits in the women with whom they came into contact.

Between May 2009 and February 2011, 211 health and family support staff working in 14 SSCCs in Southampton UK were trained in Healthy Conversation Skills, reaching approximately 70% of eligible SSCC staff in Southampton. Staff practice was assessed immediately after training, after some weeks and then a year later. After training, staff demonstrated sustained changes to the way they worked with women attending SSCCs, and were more confident in supporting lifestyle changes [29]. Changes to the way staff worked were still evident a year post-training [30]. A non-randomised controlled trial of the effect of exposure to trained staff on diet and physical activity levels in women attending SSCCs showed that the intervention had no measurable effect on women’s diet and physical activity levels, but that it may have protected against a decline in their self-efficacy and perceptions of control over life, factors known to be associated with change in both these health behaviours [31].

The story of the development of the Southampton Initiative for Health and the subsequent adoption by Southampton City Council of Healthy Conversation Skills training suggests that there are three necessary processes in translation from research to practice: building trust, matching agendas and changing culture.

### 4.1. Building Trust

The development and testing of Healthy Conversation Skills training and the construction of the Southampton Initiative for Health partnership took place over a long period. Box 2 shows the timeline from the first exploration of ideas underpinning the development of Healthy Conversation Skills to roll-out as a public health initiative, a process that took eight years. Whilst there is no suggestion that all research translation need take this long, the inclusion of this timeline is intended to illustrate the investment needed to build relationships between academic and local authority/healthcare partners and thereby to translate research findings into public health practice.

Box 2 **Timeline of events in the Southampton Initiative for Health.**

**Timeline of Events in Progress of the Partnership That Supported the Development, Testing and Implementation of Healthy Conversation Skills Training.**2004Research team made contact with SureStart Children’s Centres and began a series of focus group discussions with Centre users to explore food choices and barriers to healthy eating.2005Research team set up Food Choice Action Group including research team of psychologists, nutritionists, public health experts, statisticians, external academic advisors, members of local health authority and SureStart Children’s Centre managers.2007The research arm of the Southampton Initiative for Health formally established.Research team conducted a Nutrition and Well-being Survey in SureStart Children’s Centres with mothers. Local authority and Primary Care Trust carried out a training needs assessment of SureStart staff.2008Research team met SureStart Children’s Centre managers, health visitors and health trainers to discuss results of the Nutrition and Well-being Survey.Researchers from the University’s Medical Research Council Lifecourse Epidemiology Unit invited Southampton Primary Care Trust to discuss needs of the local area.Formal meeting was held of all local agencies from across the Primary Care Trust and City Council to discuss the research findings and how they might be used as the basis for an intervention to improve the nutritional status of local young women and their children.Southampton Initiative for Health as a formal partnership between research, policy and practice organisations was born. A programme of monthly meetings involving participating organisations was established.A focus group discussion was held with key SureStart Children’s Centres practitioners to discuss findings from discussions and survey work with women attending SureStart Children’s Centres, and to gather ideas for potential intervention points.2009Research team observed a range of SureStart Children’s Centres sessions across Southampton.Healthy Conversations Skills training was developed by the whole Southampton Initiative For Health team. Training manual was written.Research team held a pilot session of the training with SureStart Children’s Centre managers. Training manual was modified.SureStart Children’s Centres managers encouraged their staff to attend Healthy Conversation Skills training, to be delivered at the Centres. Southampton Initiative For Health and SureStart Children’s Centres worked together to recruit staff.Training began across the city.2010–2011All Healthy Conversation Skills training sessions and follow-up completed early 2011. Research team monitored fidelity of their training delivery.Research team attended a wide range of SureStart Children’s Centres sessions to observe trainees in action, evaluate competence in Healthy Conversation Skills and provide feedback.Evaluation data collected as part of trial analysed by research team.Local commissioners formally issued a contract to the regional health promotion team to deliver Healthy Conversation Skills training.Research team ‘handed over’ the intervention to be run by local services. Research team trained health promotion team in Healthy Conversation Skills.Health promotion team begins delivery of training to Primary Care Trust and city council staff in Southampton late 2010.Training sessions delivered by health promotion team observed by Southampton Initiative for Health research team to assess fidelity of intervention delivery.Evaluation data collected from health promotion team training analysed by research team.2011Southampton Initiative for Health provided all trainees with an interactive Healthy Conversation Skills newsletter.Research team conducted another round of observations of trainees in practice at SureStart Children’s Centres.2012Research team conducted focus groups with trainees to gauge feasibility and acceptability of training programme.Reports and papers produced by joint authorship representing all organisations participating in the Southampton Initiative for Health.

Partnership began in 2004 with series of focus group discussions with women attending the Centres. The focus groups identified a series of barriers and facilitators faced by young women in providing themselves and their children with a healthy diet and lifestyle. Women knew they should be feeding themselves and their children well and exercising more, but felt other aspects of life prevented them from making these healthy choices [32,33]. Any effective intervention would therefore have to focus on engaging and motivating women to overcome these barriers and make changes rather than simply informing or educating them. This was the stimulus for the development of Healthy Conversation Skills training which then took place over the next three to four years.

In 2008, SSCC managers, local agencies, members of the City Council and local public health team were invited to a formal meeting with the research team and senior representatives of the university. The meeting shared the research evidence gathered by the University of Southampton about diet and lifestyle issues for disadvantaged young women and their children in the city, and a needs assessment carried out by Public Health that showed a gap in practitioner skills in supporting behaviour change. An explicit commitment from all parties present to addressing these issues was sought. This was the point at which the partnership was formalised and support enlisted for the testing of Healthy Conversation Skills training. The partnership was then sustained by regular joint meetings and presentations. It was through ongoing communication exercises like this one that trust between the partners in the Southampton Initiative for Health was built up.

Both Rycroft-Malone et al.’s application of their Promoting Action on Research Implementation in Health Services (PARIHS) framework, and Rychetnik et al.’s presentation of their integrated framework of translation conclude that the individuals involved play an important role in translation of research into practice [34,35]. In their terms, successful translation requires ‘a planned facilitated process involving an interplay between individuals, evidence, and context’ [34]. The development of the Southampton Initiative for Health was such a process, for which the partnership between the researchers, practitioners and commissioners of services provided a forum.

### 4.2. Agenda Matching

In 2008, the university research team ran an expert focus group with SSCC practitioners and managers which made clear the amount of effort they and others expended on engaging women and children in the services they offered [21]. His was particularly true of the most disadvantaged and socially isolated families in their areas. At the same time, the research team’s observations of practice and Southampton Primary Care Trust’s needs assessment both identified a need for practitioners to be trained in skills to support lifestyle change in patients and clients [21]. The fact that the research and practice agendas matched so closely both eased the process and dictated the nature of the intervention that was developed. One of the team (JD) who had a joint appointment at the university and the primary care trust played a crucial negotiating role in the partnership. Her role enabled her to communicate directly with practitioners, researchers and local policy makers, and to provide a conduit for information between groups to ensure all agendas were met.

This process ensured that the research agenda was a good fit with the needs of practitioners, and that there was sufficient evidence that Healthy Conversation Skills training was worth testing for effectiveness. The partnership developed a plan for assessing the impact of Healthy Conversation Skills training for SSCC staff on the diets and physical activity levels of women attending SSCCs in Southampton. Figure 1 is a copy of the logic model which formed the basis for the intervention and evaluation, the findings of which are reported above.

Following the intervention study, local commissioners were shown evidence of the effect of Healthy Conversation Skills training on the competence and confidence of their staff and this led to them commissioning the training to be delivered by the regional health promotion team to all new city council staff. The research team then trained the health promotion team to deliver Healthy Conversation Skills, and conducted some initial monitoring for quality assurance purposes. The speed with which training in Healthy Conversation Skills was commissioned and the size of the area for which it was commissioned despite lack of evidence for its effectiveness in improving women’s diets and physical activity levels, illustrate the difference in agendas between the research and practice arms of the partnership. The need for training in skills to support behaviour change and evidence of the intervention’s effectiveness in doing this was sufficient for commissioners. This early adoption of a programme in the absence of conclusive evidence of effectiveness is not unique to Healthy Conversation Skills. The SureStart scheme was itself taken from pilot to full national roll-out before there was any formal evidence of its effectiveness [36].

May proposes that for an intervention to be successfully translated or ‘normalised’ it needs to be workable in everyday practice, that the social system needs to exist to support coordination of implementation, that there has to be commitment on the part of the individuals involved and that this commitment to action has to be ongoing [37]. The process of agenda matching undertaken by the Southampton Initiative for Health was an attempt to maximise this support and commitment. Experience suggests that it may be facilitated by genuine partnerships between all agents in the process.

An important factor in the translation of the intervention was the adaptability of the training programme. The resources developed by the research team to support the training were designed to be user-friendly and to allow flexibility in the training delivery around the core competencies. Clear identification of the key skills and exercises designed to promote those skills meant that the training programme could be shortened if required without losing its essential elements or integrity. The generalisability of the skills meant the training could be easily modified to suit the previous experience and roles of trainees. The intervention design was intended to be flexible enough to accommodate local variations, and retaining key concepts against which progress, delivery and effectiveness could be monitored and evaluated [38,39]. The materials were developed and tested through discussion and partnership with the local managers of health and social care services and with the managers of SSCCs. The team was conscious of the need for training and intervention protocols that could be used in real world settings. Kok et al. describe the process of intervention adaptation as a key factor in successful translation from research into practice [40]. Experience with the Southampton Initiative for Health demonstrated that the process of making necessary modifications to the intervention whilst retaining the programme’s integrity requires constant dialogue.

### 4.3. Changing Cultures

A clash of cultures between researchers and practitioners manifested itself in the early stages of intervention development. The intention of the researchers in running an expert focus group with a small group of influential practitioners was to present them with the views of the women, who used their services, to discuss with them the difficulties they experienced in maintaining a healthy diet and lifestyle and to consult them about how these findings might inform an intervention to improve the nutritional status of women and their children. Practitioners, however, were more focused on the process of engaging and working with women, and the challenges inherent in achieving this within a system that favoured short-term funding, than they were in addressing the substantive issues the research team had identified [21]. This is not surprising in retrospect, but at the time it meant that the research team were made to understand that the process of engaging both staff and women attending SSCC had to begin with where their interests actually lay, not where evidence suggested they should. The product of this realisation was that the research team adjusted their early conceptions of the intervention to one that focused on providing practitioners with additional skills to engage women in behaviour change; training that would continue to benefit them and their clients beyond the period of funding. Equally, commissioners and providers of services had to accommodate a research culture which required repeated data collection for the purposes of evaluation, a commitment of staff time and a willingness to undertake long term follow-up.

Another major learning from the Southampton Initiative for Health was about the value of ‘bottom-up’ support for interventions. Senior managers in the agencies involved in the partnership were supportive of the intervention and of the evaluation, as were front-line practitioners. Though the researchers made every effort to minimise the requirements of taking part in the study, managers of services had to be prepared to commit precious resource to support the training in Healthy Conversation Skills and evaluation of its impact. This created some tensions and may have led to less than complete recruitment of staff in SureStart Children’s Centres eligible for training. Notwithstanding the strength of the relationship between members of the partnership, there were also inevitable instances of miscommunication which had to be resolved. Fundamentally, researchers and practitioners have different cultures which include different emphases, values and language, evidence of which came from practitioner reactions to the data shared with them as part of the expert focus group discussion.

The adoption of a Healthy Conversation Skills or empowerment approach to supporting diet and lifestyle change may require change in the culture of organisations. New patterns of working and individual change in the way health and social care staff practise, have to be accommodated. It is not clear how successful the Southampton Initiative for Health was in changing organisational culture. Supporting this change demands good communication, not just a process of consultation, but a genuine partnership between all those involved in intervention implementation. Changes have to be made WITH stakeholders rather than TO stakeholders, which implies that a process of stakeholder consultation will not be enough to ensure translation. What the Southampton Initiative for Health attempted was the formation of a genuine partnership. This, rather than consultation, is likely to be key to the translation from research to public health practice.

Changing policy contexts present another challenge to the translation of research into practice. Research teams ideally need partners in public health practice to stay abreast of local policy changes. Because it is based on a broad skill set, however, Healthy Conversation Skills as an approach has the major advantage of being generalisable across changing policy contexts. The current movement in UK local government to ‘make every contact count’ is an attempt to build on the opportunity that all health and social care practitioners have to improve health through supporting behaviour change in the millions of people with whom they come into contact. It is not clear, however, that these practitioners currently have the skills to do so [26].

## 5. Conclusions

Advances in our understanding of drivers of social behaviour and of methods of supporting behaviour change can be fruitfully applied to interventions that address imperatives from developmental origins research. Training health and social care practitioners in skills to support behaviour change (Healthy Conversation Skills) represents one such intervention strategy that can be applied at scale and used to engage disadvantaged populations in diet and lifestyle change and hence translate the science of the developmental origins of health and disease into improvements in human health.

The Southampton Initiative for Health illustrates some of the opportunities and challenges there are inherent in the translation of developmental origins research into practice. The overriding feature of the Healthy Conversation Skills training intervention is that it was initiated, designed, implemented, evaluated and rolled-out in partnership with commissioners and providers of health and social care in the city of Southampton. The adaptable nature of the intervention meant that it was flexible and responsive to the changing needs of providers; these needs could be identified and communicated because of the genuine nature of the partnership.

Building a genuine partnership based on a relationship of trust takes a long time, in this case eight years, and a major commitment to fostering good communication. Experience with the Southampton Initiative for Health suggests that this commitment maybe fundamental to any community-based intervention. The value of this activity in empowering women to make better diet and lifestyle choices for themselves and their families, and empowering the health and social care workforce to offer more effective support for maternal, infant and family nutrition, is that it has potential to improve the health of generations to come.

## Figures and Tables

**Table 1:** Healthy Conversation Skills (HCS) training mapped onto Behaviour Change Techniques (cf. BCT Taxonomy v1: 93 hierarchically-clustered techniques) [26].

BCT Group No.	BCT Group Name	BCT No.	BCT	Example of Activity Component in HCS Training
1	Goals & planning	1.2	Problem-solving	Prompt trainees to generate/select strategies to overcome barriers & increase facilitators to using HCS in routine practice; includes “relapse prevention” & “coping planning”.
		1.6	Discrepancy between current behaviour & goal	Draw trainee’s attention to discrepancies between current practice and plans/goals to incorporate HCS into practice.
3	Social support	3.2	Social support (practical)	Group training & pair work provides practical support (listening & sharing tips) for practising HCS in the training & later in the workplace.
		3.3	Social support (emotional)	Group training & pair work provides emotional support (encouragement/praise) for practising HCS in a safe/comfortable environment.
4	Shaping knowledge	4.1	Instruction on how to perform the behaviour	Skills training, including exploration & agreement on how to develop questions, support SMARTER planning etc.
		4.2	Information on antecedents	Review with trainees what predicts behaviour (& possible relapse to old behaviour patterns), e.g., when under time pressure might revert to telling or suggesting.
6	Comparison of behaviour	6.1	Demonstration of the behaviour	Trainees growing awareness that HCS is being modelled by the trainer in all activities, & increasingly by other trainees in real/role play activities.
7	Associations	7.1	Prompts & cues	Resources provided in the training room to prompt use of the skills throughout the training; hand-outs to be used by trainees in their workplace to remind to use HCS.
8	Repetition & substitution	8.1	Behavioural practice/rehearsal	Prompt practice of HCS in training room by providing numerous opportunities.
		8.3	Habit formation	Prompt practice of HCS in real world, by encouraging action-planning and problem-solving.
		8.6	Generalisation of a target behaviour	If trainee has used HCS with friend/relative, encourage to try out skills in workplace.
6	Comparison of behaviour	6.2	Social comparison	Opportunities to compare own practice & experiences with others, including pre-training behaviour and then increasing use of HCS.
13	Identity	13.3	Incompatible beliefs	Draw attention to discrepancy between current/past practice and view of self as effective health practitioner. Embedding HCS is one way to reduce these incompatible beliefs & discrepancies.

HCS = Healthy Conversation Skills; BCT = Behaviour Change Technique; SMARTER = Specific, Measurable, Action-orientated, Realistic, Timed, Evaluated, Reviewed goal-setting and planning.
